# Evaluation of Prognostic Factors for Survival in Transverse Colon Cancer

**DOI:** 10.3390/cancers12092457

**Published:** 2020-08-30

**Authors:** Michela Roberto, Giulia Arrivi, Francesca Lo Bianco, Stefano Cascinu, Fabio Gelsomino, Francesco Caputo, Krisida Cerma, Michele Ghidini, Margherita Ratti, Claudio Pizzo, Corrado Ficorella, Alessandro Parisi, Alessio Cortellini, Federica Urbano, Maria Letizia Calandrella, Emanuela Dell’Aquila, Alessandro Minelli, Claudia Angela Maria Fulgenzi, Ludovica Gariazzo, Andrea Montori, Emanuela Pilozzi, Marco Di Girolamo, Paolo Marchetti, Federica Mazzuca

**Affiliations:** 1 Department of Clinical and Molecular Medicine, Oncology Unit, Sant’ Andrea Hospital, Sapienza University of Rome, 00187 Rome, Italy; michela.roberto@uniroma1.it (M.R.); giulia.arrivi@uniroma1.it (G.A.); francesca.lobianco@uniroma1.it (F.L.B.); ludovicagariazzo@gmail.com (L.G.); paolo.marchetti@uniroma1.it (P.M.); 2 Division of Oncology, Department of Oncology and Hematology, University Hospital of Modena, 41125 Modena, Italy; cascinu@yahoo.com (S.C.); fabiogelsomino83@yahoo.it (F.G.); francesco1990.caputo@libero.it (F.C.); kridi90@gmail.com (K.C.); 3 Oncology Unit, Oncology Department, ASST of Cremona, 26100 Cremona, Italy; micheleghidini@outlook.com (M.G.); margherita.ratti@studenti.unipr.it (M.R.); claupizz1987@gmail.com (C.P.); 4 Medical Oncology, St. Salvatore Hospital, University of L’Aquila, Department of Biotechnological and Applied Clinical Sciences, University of L’Aquila, 67100 L’Aquila, Italy; corrado.ficorella@univaq.it (C.F.); alexparis@hotmail.it (A.P.); alessiocortellini@gmail.com (A.C.); 5 Department of Radiology, Oncology and Pathology, Policlinico Umberto I, Sapienza University of Rome, 00185 Rome, Italy; federica.urbano@uniroma1.it (F.U.); marialetiziacalandrella@gmail.com (M.L.C.); 6 Medical Oncology Department, Campus Bio-Medico University of Rome, 00128 Rome, Italy; e.dellaquila@unicampus.it (E.D.); a.minelli@unicampus.it (A.M.); c.fulgenzi@unicampus.it (C.A.M.F.); 7 Department of Clinical and Molecular Medicine, UOC Anatomia Patologica, Sant’ Andrea Hospital, Sapienza University of Rome, 00187 Rome, Italy; andrea.montori@uniroma1.it (A.M.); emanuela.pilozzi@uniroma1.it (E.P.); 8 Department of Radiology, Sant’Andrea University Hospital, 00187 Rome, Italy; marco.digirolamo@uniroma1.it

**Keywords:** TCC, sidedness, prognostic factors, tumor grade, BRAF

## Abstract

**Simple Summary:**

Transverse colon cancer (TCC) is mostly included among right-sided colon cancer, and sometimes even excluded at all, thus it is not completely clear if they present total similarities with right-sided ones or if they have their own specific features. With a median follow-up of 34 months, we concluded that TCC shares some clinicopathological characteristics with left-sided colon cancer and many others with the right-sided ones, but only poorly/undifferentiated tumor grade and BRAF V600E mutation are independent prognostic factors for survival, regardless of tumor stage. The present study provides more insightful knowledge of clinicopathological characteristics of TCC patients, emphasize the role of BRAF mutation since the early stage of disease and lay the basis for new treatment algorithms in this specific setting of colon cancer.

**Abstract:**

*Background:* Although most of the analyses included transverse colon cancers (TCC) among right colon cancer (RCC), it is not completely clear if they present total similarities with RCC or if they have their specific features. Therefore, we present an observational study to evaluate clinicopathological characteristics and survival data of patients with TCC. *Methods:* We retrospectively reviewed 450 RCC, of whom 97 stages I–IV TCC were included in this multicenter study; clinicopathological and molecular parameters were analyzed to identify prognostic factors for disease-free survival (DFS) and overall survival (OS). *Results:* Most of TCC cases were male (61%), with ≤70 years old (62%), and good performance status (ECOG PS 0, 68%). According to WHO classification, 41 (49%) and 40 (48%) tumors were classified as well to moderate and poorly/undifferentiated respectively, regardless of mucinous component (30%). About molecular data, 8 (26%), 45 (63%), and 14 (24%) were MSI-H, KRAS wild-type, and BRAF V600E mutant, respectively. With a median follow-up of 34 months, there were 29 and 50 disease recurrences and deaths respectively. Charlson comorbidity index ≥5 was a significant prognostic factor for DFS (HR = 7.67, 95% CI 2.27–25.92). Colon obstruction/perforation (HR = 2.65, 95% CI 1.01–7.01), and BRAF mutant (HR = 3.03, 95% CI 0.97–9.50) cases showed a worst, despite not statistically significant, DFS. Whereas for OS, at the multivariate model, only tumor grade differentiation (HR = 5.26, 95% CI 1.98–14.01) and BRAF mutation status (3.71, 95% CI 1.07–12.89) were independent prognostic factors. *Conclusions:* Poorly/undifferentiated tumor grade and BRAF V600E mutation are independent prognostic factors for OS in TCC. Further prospective clinical trials are needed to better define TCC treatment in order to improve patient outcome.

## 1. Introduction

The right-sided and left-sided primary colon tumors are defined as those originating proximally or distally to the splenic colic flexure, based on the different embryological origin from the midgut and hindgut, respectively. However, the difference between these tumors can be attributed to a combination of anatomical and developmental origin, as well as distinct carcinogenic factors [1]. A number of studies have shown that patients affected with right colon cancer (RCC) are predominantly female and older than those affected by left colon cancer (LCC) [2]. Furthermore, proximal tumors tend to involve bulky, exophytic, polypoid lesions growing into the colon lumen and are associated with advanced stages, increased tumor size, poorly differentiated grade, and different molecular biological tumor patterns [3]. In contrast, the characteristics of distal localizations tend to involve infiltrating, constricting lesions encircling the colorectal lumen and causing obstruction [4]. About the site of metastases, left-sided tumors are associated with liver and lung metastases, instead right-sided seems to have more peritoneal involvement [3], historically correlated to worst outcome. According to recent post-hoc analysis of large clinical trials, sidedness is recently proposed as a surrogate prognostic and predictive marker of survival in colon cancer [5,6,7,8]. As a matter of fact, colorectal cancers (CRCs) may have different histological and genetic characteristics as well as a different outcome in terms of disease progression and overall survival based on the specific tumor location [6,7,8]. RCCs show sessile serrated adenomas or mucinous adenocarcinomas and tend to have more microsatellite instability-high (MSI-high), while LCC show tubular, villous or typical adenocarcinomas and present a genomic make-up of chromosomal instability-high (CIN-high) [9]. Thus, increasing evidence suggests that a continuum of characteristics and behaviors can be described throughout different colorectal segments, from the caecum to extraperitoneal rectum, rather than a simplistic dichotomic distinction.

Transverse colon originates embryologically 2/3 from the midgut and 1/3 from hindgut, thus transverse colon cancers (TCC), defined as tumors originating distally to the hepatic flexure and proximally to the splenic flexure, share some characteristics with RCC and others with LCC [9].

Properly TCC is a rare condition that accounts for 10% of all colon cancer. Although most of the analyses included tumors originating from transverse colon among RCC, and sometimes even excluded at all [10,11,12], it is not completely clear if they present total similarities with right-sided ones or if they have their own specific features. Like RCC, TCC shows poor prognosis, it occurs more frequently in advanced stages [13] and presents a microsatellite instability status in a consistent number of cases [14]. On the contrary, rather like LCC, the RAS/BRAF wild type TCC cases were more responsive to anti-EGFR agents [10]. Therefore, TCC lies in a continuum of different diseases from right to left side of the colon and unlike both RCC and LCC shows neither reliable prognostic factors nor specific outcome data yet.

Given these considerations, we present an observational study that examined in patients with stage I-IV TCC, several clinicopathologic features, including KRAS, BRAF, and MSI, to evaluate any relationship between their expression and clinical outcome.

## 2. Patients and Methods

### 2.1. Patients

We retrospectively reviewed clinical records of a total of 450 patients affected with RCC, whose data were consecutively collected from 2007 to 2018 in 5 Italian centers, and those patients reported a stage I–IV TCC were analyzed in this study. TCC was defined as colon tumor originating distally to the hepatic flexure and proximally to the splenic flexure. For each patient demographics data (sex, age, comorbidities, grouped according to Charlson comorbidity index (CCI), Eastern Cooperative Oncology Group (ECOG) performance status at the time of primary diagnosis), symptoms at the clinical onset, and both histopathological (pT, pN, the grade of differentiation, mucinous component, and lymphovascular/perineural invasion) and molecular (KRAS, BRAF V600E, and MSI) features were retrieved. The study was conducted in accordance with the Declaration of Helsinki and all patients signed informed consent for scientific research purpose at the first oncological visit. Since the observational retrospective nature of the study, we just sent the local ethical committee (Comitato Etico Sapienza Università di Roma, Rome, Italy) a notification (normative ref. GU della Repubblica Italiana n.76 of 31 March 2008).

### 2.2. Statistical Analysis

Outcome variables were disease-free survival (DFS), defined as the time between diagnosis and disease recurrence or development of distant metastasis, and overall survival (OS), defined as the time between diagnosis and death for any cause. The χ^2^–test and *t*-test for unpaired data were applied to compare frequencies and means, respectively. The interaction among clinicopathologic parameters was first analyzed using univariate logistic regression and then, those statistically significant parameters were compared by multivariate analysis. Survival curves were estimated using the Kaplan–Meier method and the log-rank test was used for the difference assessment. A multivariate Cox-proportional hazard model was used to identify independent prognostic factors for overall survival. SPSS statistical software, Version 24 (SPSS Inc. Chicago, IL, USA) was used.

## 3. Results

### 3.1. Frequency and Associations of Clinicopathologic Parameters

Overall, 97 patients affected with any stage TCC were enrolled in this study. Their clinicopathological features were reported in Table 1. Most of TCC cases were male (61%), with ≤70 years old (62%), and good performance status (ECOG PS 0, 68%). The tumors were more frequently pT3 (73%) than pT4 (21%), with lymphovascular and/or perineural invasion (63%). According to WHO classification, 41 (49%) and 40 (48%) tumors were classified as well to moderate (G1-G2) and poorly/undifferentiated (G3) respectively, regardless of mucinous component (30%). Forty-eight (49%) cases occurred in the advanced stage. Taking into account molecular data, 8 (26%), 45 (63%), 14 (24%) were MSI-H, KRAS wild-type, and BRAF V600E mutant, respectively. Most patients (86%) underwent surgery of primary tumor and among stage II–III patients, 30 (61%) received adjuvant chemotherapy.

### 3.2. Survival Analysis

**Figure 1:**
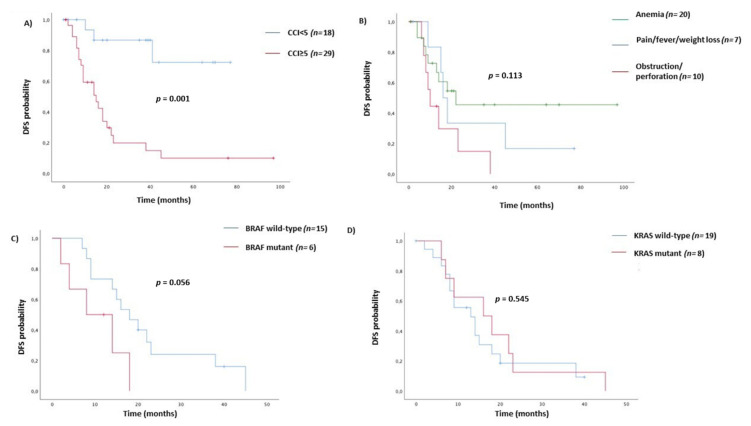
Kaplan–Meier curves for DFS according to Charlson Comorbidity Index (**A**), clinical onset (**B**), BRAF status (**C**), and KRAS status (**D**).

**Figure 2:**
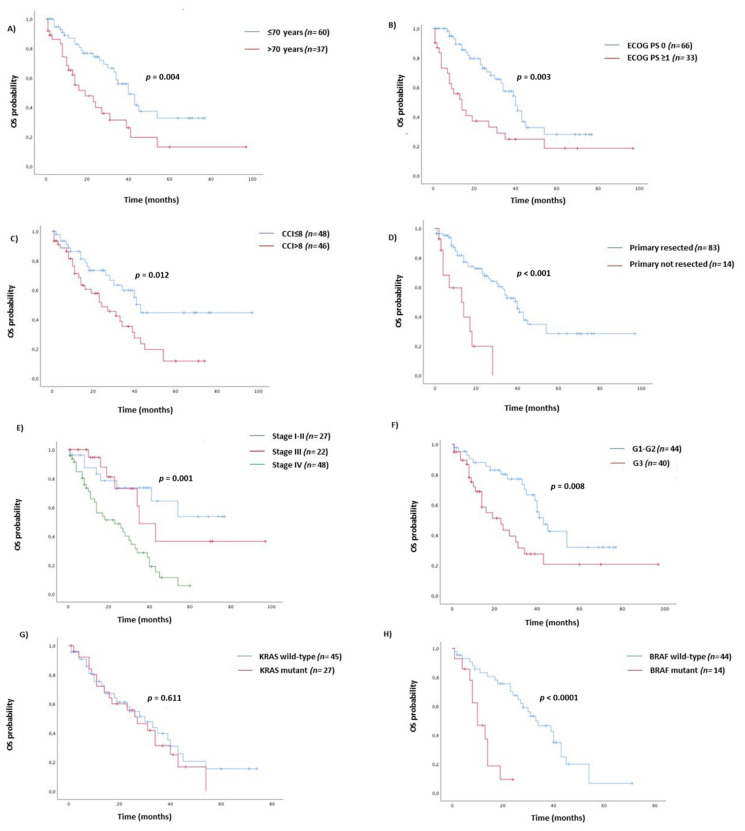
Kaplan–Meier curves for OS according to age (**A**), ECOG PS (**B**), Charlson Comorbidity Index (**C**), primary tumor surgery (**D**), TNM Stage (**E**), tumor grade (**F**), KRAS status (**G**), and BRAF status (**H**).

**Figure 3:**
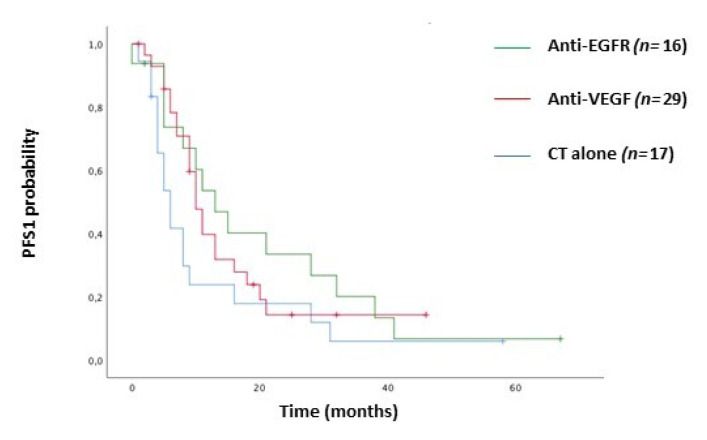
Kaplan–Meier curves for progression-free survival (PFS) according to first-line treatment.

With a median follow-up of 34 months (95% CI 23.42–44.58), there were 29/49 (59%) disease recurrences and 50/97 (51%) death events. In the adjuvant setting population (*n* = 49), no variable but for CCI ≥ 5 (HR = 7.67, 95% CI 2.27–25.92), was a significant prognostic factor for DFS. However, obstruction/perforation (HR = 2.65, 95% CI 1.01–7.01), and BRAF mutant (HR = 3.03, 95% CI 0.97–9.50) cases showed a worst, despite not statistically significant, DFS. (Table 2) (Figure 1A–C). According to prognostic TNM staging system, stage III (HR = 6.57, 95% CI 2.26–8.48) and stage IV (HR = 7.38, 95% CI 4.31–8.80) were both significantly correlated with overall survival (Table 2). In the univariate analysis of the whole study population, age ≥70 years (HR = 2.27, 95% CI 1.29–3.98), ECOG PS ≥ 1 (HR = 2.36, 95% CI 1.34–4.13), CCI ≥ 8 (HR = 2.11, 95% CI 1.18–3.80), primary tumor resected (HR = 0.22, 95% CI 0.10–0.47), pN2 (HR = 1.65, 95% CI 1.07–2.54), G3 tumor grade (HR = 2.29, 95% CI 1.24–4.22), BRAF V600E mutation (HR = 5.16, 95% CI 2.20–12.10) were those variables that significantly predict overall survival (Table 2). (Figure 2A–H) However, in a multivariate model including age, ECOG PS, CCI, primary tumor resected, and stage, only tumor grade (HR = 5.26, 95% CI 1.98–14.01) and BRAF mutation status (3.71, 95% CI 1.07–12.89) were independent prognostic factors for OS (Table 2) (Figure 2F,H). Although pN was significantly associated with survival in the univariate analysis, it was not included in the multivariate model, because of pN covariates linearly with TNM stage III. Treatment options for stage IV patients in first-line setting included 5 Fluorouracil-based chemotherapy alone (29%), eventually added to a targeted anti-vascular endothelial growth factor (anti-VEGF, 45%) or anti-epidermal growth factor receptor (anti-EGFR, 26%) drugs according to the KRAS/NRAS tumor molecular profile. The objective response rate was 33%, 59%, and 54%, respectively. With a median follow up of 50 months, the median progression-free survival (PFS) at first line was 6 v 10 v 13 months (HR = 0.73, 95% CI 0.50–1.07; *p* = 0.107) (Figure 3) and OS was 16 v 27 v 30 months (HR = 0.85, 95% CI 0.58–1.25; *p* = 0.424) with chemo alone, plus anti-VEGF and anti-EGFR targets, respectively.

### 3.3. Correlations between Tumor Grade and BRAF Status with Clinicopathologic Parameters

Poorly differentiated tumor was significantly associated with advanced age (*p* = 0.005), poor ECOG PS (*p* = 0.004), lympho-vascular/perineural invasion (*p* = 0.042) and BRAF status (*p* = 0.003) (Table 3).

As well, BRAF mutant status was significantly associated with advanced age (*p* = 0.020), and poor ECOG PS (*p* = 0.004). BRAF and KRAS mutations were mutually exclusive (*p* = 0.001) (Table 3). However, at multivariate analysis for OS, including age and ECOG PS, only tumor grade and BRAF status resulted independent prognostic factors for survival (Table 2).

## 4. Discussion

This study analyzed clinicopathological data of patients affected with any stage TCC and identify tumor grade and BRAF status as independent prognostic factors for survival. Although several limitations including the retrospective analysis, possible selection biases, and the relatively small sample size, according to the lower incidence of TCC among all CRC diagnoses, the number of TCC patients included in this study is consistent with that reported in literature data [10,11,12,13,14].

Most published studies have used to consider TCC as part of the RCC, although tumors arising from the transverse colon, according to its embryological origins, may share characteristics with right colon cancer (RCC) as well as with left colon cancer (LCC) [1,15,16,17]. Thus, it is still not completely clear if TCC behavior is more like to right-sided rather than left-sided ones or if it has its clinicopathological features and a different clinical outcome.

Until now, TCCs are mostly included among right-sided tumors or often excluded from large prospective randomized trials because of their complexity [11,12].

As previously defined, the sidedness may be considered as a surrogate prognostic and predictive marker for colorectal cancer patients [6,7,8]. The different epidemiological and clinicopathological characteristics of CRCs based on their anatomical location, is supported also by a different pattern of gene expression profile from ascending to descending colon [9]. Moreover, the mutation pattern varies dramatically within the side, and mainly within tumors of the same side but at different locations. When the mutation clusters of different locations—cecum, ascending colon, hepatic flexure, and transverse colon—were compared with each other, strong trends toward differing cluster prevalence was highlighted among these right-sided locations. Instead, in LCC, there was no difference in mutation cluster between splenic flexure, descending colon, sigmoid colon, rectosigmoid colon, and rectum when compared. An additional direct comparison between transverse colon tumors to LCC and RCC showed that transverse colon tumors differed from right-sided but not from left-sided locations [14].

Four consensus molecular subtypes (CMSs) are described in CRCs according to various key features: CMS1 (MSI Immune, 14%), enriched for MSI-high and BRAF mutation; CMS2 (Canonical, 37%) tumors, epithelial, chromosomally unstable, are marked by WNT and MYC signaling pathway activation; CMS3 (Metabolic, 13%) malignancies presented with epithelial features and apparent metabolic dysregulation; CMS4 (Mesenchymal, 23%) that exhibited a prominent stromal component, angiogenesis and transforming growth factor-β (TGFβ) activation [18,19]. Both CMS1 and CSM3 are predominantly represented in RCCs but there is heterogeneity among RCC itself. Indeed, despite right-sided CRCs are enriched mostly in CMS1 and CMS3, all 4 CMSs are represented among right-sided CRC [18]. Contrariwise LCC was characterized widely by CMS2 and in a smaller percentage by CMS4 [18,20]. These differences in molecular profiling suggest that the transverse location differs from other right and left-sided locations and attest that, because of the complexity and heterogeneity of TCC itself, the current right/left classifications may not fully explain CRCs biology.

Thus, by considering TCC as a single entity, we conducted a retrospective analysis of 97 stages I-IV TCC from a multi-institutional database to evaluate whether a precise definition of tumor location may provide more insightful knowledge of clinicopathological characteristics of TCC and to identify any correlations to patient’s outcome.

In our analysis, the most of TCC cases were male (61%), with a median age of 68 years (range 36–90), and good performance status (ECOG PS 0, 68%). In literature, it has been observed that male sex is more frequently associated with left colon cancers than right colon cancers [2,21,22]. The majority of male sex in our casuistic could be explained by the more representation of those TCC which arises from hindgut rather than those arises from midgut (57, (59%), and 40, (41%) respectively).

On the contrary, most patients presented a delayed diagnosis with iron deficiency anemia (30%) and an advanced stage of disease (3 (3%) v 24 (25%) v 22 (23%) v 48 (49%) stage I, II, III, and IV, respectively), more close to the typical RCCs clinical presentation [4,23].

In our study, 28 (30%) patients reported a mucinous histology tumor. This percentage is close to that observed in large population-based studies in which mucinous histology accounts for 19% in the right-sided colon cancer rather than 3.9% in the overall CRC [24].

We know that approximately 30% of all RCC are mismatch repair defective as recognized by the presence of MSI-H phenotype, whereas only 2% of LCC show the MSI-H phenotype [25,26]. In our study 26% of cases were MSI-H. These data suggest that given the different genomic profiles of RCC compared to LCC, transverse colon is more like RCC in terms of histology and immune-phenotype: Indeed, the CMS1 molecular subtype, characterized by MSI-H expression, is more represented in RCC [20]. These results pave the way for considering the use of immune checkpoint inhibitors in TCC patients with MSI-H phenotype since the first line of treatment and eventually to avoid useless 5-fluorouracil based chemotherapy [27,28].

From a molecular point of view, 45 (63%) and 14 (24%) patients harbored KRAS wild-type and BRAF mutant tumors, respectively. The high representation of the KRAS wild-type is more consistent with the LCC molecular profile rather than that reported in RCC [11,14].

On the other hand, the rate of BRAF mutation (24%) was more similar to RCC and consistent with data of Loree et al. [14] that showed that BRAFV600E mutations fall moving distally, from 10% of cecal to 16% of ascending colon, and 22% of hepatic flexure tumors, to demonstrate the complexity of transverse location.

To further confirmation of literature data [14], by dividing transverse colon in 2/3 proximal and 1/3 distal, we reported MSI-H phenotype in 4 (40%) of 2/3 proximal TCCs and 4 (19%) of 1/3 distal TCCs, respectively. KRAS wild type was mostly reported in distal localizations with 30 (73%) cases, instead only 15 (48%) cases in proximal ones. About BRAFV600E mutations, we highlighted 3 (12%) cases in 2/3 proximal versus 11 (32%) in 1/3 distal (Table S1).

Thus, transverse colon, usually classified as right-sided in most subgroup analyses of randomized trials, due to its heterogeneous molecular landscape, should be considered as a different clinicopathological entity over the dichotomic definition right/left CRC. Indeed, according to the retrospective study by Cremolini et al. [10] our patients with stage IV, RAS/BRAF wild-type, molecular profile have benefitted from anti-EGFR-based chemotherapy (Figure 3), as opposed to RCC cases overall [4].

With a median follow-up of 34 months, 59% of patients affected with stage II-III TCC experienced a disease recurrence. In this adjuvant setting (*n* = 49), no variable but for CCI ≥ 5 (HR = 7.67, 95% CI 2.27–25.92), was a significant prognostic factor for DFS.

As previously described, the presence of multiple comorbidities negatively impacts on CRC patient survival [29,30,31]. Moreover, a recent metanalysis [32] demonstrated that CCI was a comorbidity index significantly related to mortality risk in those colon cancer patients with many comorbidities: Patients with CCI 1–2 and CCI ≥ 3 had a 1.5 and over 2 times higher mortality risk, respectively than those without comorbidity. Furthermore, three cohort studies confirmed a significant correlation between comorbidity and poor DFS [33,34,35]. According to these results, at our univariate analysis for DFS, CCI ≥ 5 resulted to be significantly related to a higher risk of recurrence. However, since none of the other examined factors resulted to be statistically significant correlate to DFS, a multivariate analysis was not performed. Therefore, we cannot conclude that CCI was an independent prognostic factor of DFS in TCC patients.

According to other studies [36,37], our patients whose tumor occurred with obstruction or perforation showed a worse DFS compared with the other tumor manifestations, although with a trend towards significance (HR = 2.65, 95% CI 1.00–7.01, *p* = 0.050).

The prognostic role of mucinous histology is still debated. Conflicting results are found in the literature regarding the prognosis and survival of mucinous CRC: A large Europe population-based analysis demonstrated that mucinous histology in colorectal adenocarcinoma had no negative impact on survival [38]; as well, another study demonstrated that mucinous histology did not show any significant correlation with the prognosis of stage II and III CRC [39]. In agreement with these results, our study showed that mucinous histology in transverse adenocarcinoma did not correlate with the patient’s outcome. It could be explained by the low rate of G3 tumors among mucinous adenocarcinoma (*n* = 15, 37%) nonetheless by our high representation of mucinous histology like RCC cases generally. Thus, since we analyzed a homogenous casuistic of TCC, the prognostic role of mucinous histology when it was usually compared between LCC versus RCC, could be lost. Indeed, as reported in the study of Wang ZX et al., the prognostic value of mucinous histology differed according to sidedness, and vice versa [40].

About OS, in the multivariate analysis of the whole study population, only tumor grade (HR = 5.26, 95% CI 1.98–14.01) and BRAF status (3.71, 95% CI 1.07–12.89) were independent prognostic factors. These results are supported by those published studies in which poor histological differentiation is associated, not only with advanced T stage and the presence of lymph node involvement [23], but also directly with unfavorable clinical outcome [41] and cause-specific survival [42].

According to other studies on metastatic colorectal cancer (mCRC) [3,43,44,45], TCC patients harboring BRAFV600E mutation showed a significantly worse OS than BRAF wild type. (Table 2) As we know by literature, BRAF mutation represents the main negative prognostic factor for mCRC, regardless of sidedness and other molecular factors [43,44] but this negative effect on prognosis seems to be more evident in RCC rather than LCC [3,45].

Furthermore, our results revealed that poorly differentiated tumor was significantly associated with BRAF status, as well as with advanced age, poor ECOG PS, and lymphovascular/perineural invasion. (Table 3) In turn, BRAF mutations are associated with distinct unfavorable clinicopathological characteristics, including poor differentiation as reported also in some published studies [46,47,48]. From our data, BRAF mutation showed a trend towards poor DFS, despite not statistically significant (Table 2). The possible role of BRAF mutation as an independent negative prognostic factor in stage II and III CRC in addition to stage IV has previously reported in limited cohort studies [49]. However, we found BRAF status definition only in 21 out of 49 stages I-III patients. Thus, the limited sample size does not allow to draw any definitive conclusion in term of DFS. About OS data, the prospective mutational analysis from resected CRC patients included in the PETACC-3 study has revealed poorer OS for those patients harboring a BRAF mutation, with an even greater impact in stage III [50]. Accordingly, by considering the whole study population, BRAF mutation resulted to be an independent prognostic factor for OS, regardless of the TNM stage. Thus, the value of BRAF mutation as an additional risk factor in stage III other than stage IV TCC could better discriminate, between patients with a worse prognosis, who could benefit from target treatments since the early stage of the disease.

## 5. Conclusions

The present study provides more insightful knowledge of clinicopathological characteristics of TCC patients and emphasizes the role of BRAF mutation since the early stage of disease as putative predictive factor of response to targeted treatment in those patients with worse prognosis. Thus, we encourage more clinical trials including TCC patients, and lay the basis for new treatment algorithms in this specific setting of colon cancer.

## Figures and Tables

**Table 1:** Clinicopathologic features (valid cases and percentages).

Total		N.	%
		97	100
Age	68 (36–90)		
Median (range)			
≤70 years		60	62
>70 years		37	38
Sex			
Male		59	61
Female		38	39
Transverse locations			
2/3 proximal		40	41
1/3 distal		57	59
ECOG PS			
0		66	68
≥1		31	32
Charlson Comorbidity Index (*n* = 94)			
≤8		48	51
>8		46	49
Tumor onset (*n* = 73)			
Anemia		28	32
Obstruction/Perforation		22	30
Pain/fever/weight loss		23	38
Surgery of primary tumor			
Yes		83	86
Not		14	14
AJCC TNM stage			
I		3	3
II		24	25
III		22	23
IV		48	49
Pathological Tumour size (*n* = 75)			
T1		3	4
T2		1	1
T3		55	73
T4		16	21
Pathological Node status (*n* = 75)			
N0		28	37
N1		24	32
N2		23	31
Mucinous Histology (*n* = 85)		28	30
Lymphovascular/Perineural invasion (*n* = 62)			
Not		23	37
Yes		39	63
Tumour differentiation (*n* = 84)			
G1		3	3
G2		41	49
G3		40	48
Microsatellite Instability (*n* = 31)			
MSS		23	74
MSI-H		8	26
KRAS status (*n* = 72)			
Wild-type		45	63
Mutant		27	37
BRAF status (*n* = 58)			
Wild-type		44	76
Mutant		14	24
Adjuvant chemotherapy (*n* = 49)			
Yes		30	61
Not		19	39

**Table 2:** Univariate and multivariate analyses of disease free survival (*n* = 49) and overall survival in the study cohort (*n* = 97).

Variables	Disease Free Survival		Overall Survival			
	Univariate		Univariate		Multivariate	
	HR (95%CI)	p *	HR (95%CI)	p *	HR (95%CI)	p *
Age						
<70	Ref	0.210	Ref		Ref	
≥70	1.63 (0.75–3.50)		2.27 (1.29–3.98)	0.004	1.49 (0.50–4.44)	0.466
Sex						
Female	Ref	0.386	Ref			
Male	0.71 (0.32–1.54)		0.93 (0.52–1.22)	0.828		
Transverse location						
2/3 proximal	Ref		Ref			
1/3 distal	0.56 (0.26–1.20)	0.138	0.85 (0.49–1.49)	0.587		
ECOG PS						
0	Ref	0.863	Ref		Ref	
≥1	1.07 (0.47–2.64)		2.36 (1.34–4.13)	0.003	1.2 (0.31–2.26)	0.707
CCI						
<5	Ref		** Ref		Ref	
≥5	7.67 (2.27–25.92)	0.001	2.11 (1.18–3.80)	0.012	1.56 (0.25–1.63)	0.348
Tumor Onset						
Anemia	Ref		Ref			
Pain/fever/weight loss	1.35 (0.45–4.07)	0.584	1.23 (0.55–2.76)	0.602		
Obstruction or perforation	2.65 (1.00–7.01)	0.050	1.96(0.87–4.38)	0.101		
Primary tumor resected						
No	NA		Ref		Ref	
Yes			0.22 (0.10–0.47)	<0.001	0.25 (0.05–1.27)	0.097
AJCC 7th Stage						
I–II	Ref		Ref		Ref	
III	1.13 (0.52–2.45)	0.757	6.57 (2.26–8.48)	0.010	1.04 (0.32–3.27)	0.957
IV	NA		7.38 (4.31–8.80)	0.001	2.73 (0.95–7.63)	0.061
pT						
1–3	Ref	0.553	Ref			
4	1.38 (0.47–3.99)		1.64 (0.73–3.68)	0.229		
pN						
0	Ref		Ref			
1	1.02 (0.37–2.71)	0.229	1.54 (0.59–4.04)	0.377		
2	1.25 (0.42–3.80)	0.632	1.65 (1.07–2.54)	0.022		
Mucinous Histology						
Not	Ref	0.095	Ref			
Yes	0.43 (0.16–1.15)		1.17 (0.64–2.13)	0.603		
Grade						
G1–G2	Ref	0.671	Ref		Ref	
G3	1.20 (0.52–2.79)		2.29 (1.24–4.22)	0.008	5.26 (1.98–14.01)	0.001
LV/Pn invasion						
Not	Ref	0.925	Ref			
Yes	1.04 (0.41–2.66)		2.27 (1.01–5.14)	0.050		
KRAS status						
Wild-type	Ref	0.545	Ref			
Mutant	1.32 (0.53–3.28)		1.16 (0.64–2.12)	0.611		
BRAF status						
Wild-type	Ref		Ref		Ref	
Mutant	3.03 (0.97–9.50)	0.056	5.16 (2.20–12.10)	<0.0001	3.71 (1.07–12.89)	0.039
MMR status						
MSS	Ref		Ref			
MSI	1.80 (0.44–7.32)	0.410	1.44 (0.38–5.51)	0.592		
Chemotherapy						
Yes	Ref		Ref			
No	1.28 (0.58–2.86)	0.532	1.43 (0.52–3.97)	0.484		

HR, hazard ratio; CI, confidence interval; Ref, reference; NA, not applicable; LV, lymphovascular, Pn, perineural. * *p* < 0.05. is considered statistically significant. ** CCI calculated with a cut off ≤8, since metastatic patients are included in.

**Table 3:** Correlation between tumor grade and BRAF status with clinic-pathological variables.

Variables	Tumor Grade			BRAF		
	G1–G2 (*n* = 44)	G3 (*n* = 40)	*p*	Wilde-type (*n* = 44)	Mutant (*n* = 14)	*p*
	*n*(%)	*n*(%)		*n*(%)	*n*(%)	
Age						
≤70	32 (73)	17 (42)		31 (70)	5 (36)	
>70	12 (27)	23 (58)	0.005	13 (30)	9 (64)	0.020
Sex						
Female	19 (43)	15 (37)		18 (41)	7 (50)	
Male	25 (57)	25 (63)	0.596	26 (59)	7 (50)	0.550
ECOG PS						
0	36 (82)	21 (52)		34 (77)	5 (36)	
≥1	8 (18)	19 (48)	0.004	10 (26)	9 (64)	0.004
Tumor Onset Pain/fever/weight loss	8 (25)	10 (32)	0.712	13 (38)	3 (27)	0.798
Occlusion or perforation	11 (34)	8 (26)		10 (29)	4 (36)	
Anemia	13 (41)	13 (42)		11 (32)	4 (36)	
AJCC 7th Stage						
I–II	17 (39)	9 (22)	0.174	8 (18)	1 (7)	0.226
III	8 (18)	13 (32)		7 (16)	5 (36)	
IV	19 (43)	18 (45)		29 (66)	8 (57)	
Mucinous H						
Not	34 (79)	25 (62)	0.096	29 (69)	9 (64)	0.741
Yes	9 (21)	15 (37)		13 (31)	5 (36)	
pT						
1–3	30 (77)	26 (79)	0.850	24 (77)	7 (78)	0.982
4	9 (23)	7 (21)		7 (23)	2 (22)	
pN						
0	17 (44)	10 (30)	0.294	8 (26)	1 (11)	0.323
1	13 (33)	10 (30)		11 (35)	2 (22)	
2	9 (23)	13 (40)		12 (39)	6 (67)	
LV/Pn invasion						
Not	15 (50)	8 (25)		7 (26)	3 (30)	
Yes	15 (50)	24 (75)	0.042	20 (74)	7 (70)	0.804
KRAS status						
Wild-type	20 (64)	19 (68)	0.787	23 (52)	14 (100)	
Mutant	11 (36)	9 (32)		21 (48)	0	0.001
BRAF status						
Wild-type	24 (92)	12 (55)				
Mutant	2 (8)	10 (45)	0.003			
MMR status						
MSS	13 (87)	9 (69)	0.262	14 (87)	3 (75)	0.531
MSI	2 (13)	4 (31)		2 (13)	1 (25)	

LV, lymphovascular, Pn, Perineural.

## Supplementary Materials

The following are available online at https://www.mdpi.com/2072-6694/12/9/2457/s1, Table S1: Clinicopathologic features (valid cases and percentages).
