# Supplementary material for: Evaluation of Prognostic Factors for Survival in Transverse Colon Cancer
Source: Cancers (Basel). 2020 Aug 30;12(9):2457. doi: 10.3390/cancers12092457 (PMC7563638; doi:10.3390/cancers12092457)
Supplement: Supplementary file 1 [file cancers-12-02457-s001.pdf]

Article

Evaluation of Prognostic Factors for Survival in Transverse Colon Cancer

Michela Roberto, Giulia Arrivi, Francesca Lo Bianco, Stefano Cascinu, Fabio Gelsomino, Francesco Caputo, Krisida Cerma, Michele Ghidini, Margherita Ratti, Claudio Pizzo, Corrado Ficorella, Alessandro Parisi, Alessio Cortellini, Federica Urbano, Maria Letizia Calandrella, Emanuela Dell'aquila, Alessandro Minelli, Claudia Angela Maria Fulgenzi, Ludovica Gariazzo, Andrea Montori, Emanuela Pilozzi, Marco Di Girolamo, Paolo Marchetti and Federica Mazzuca

Table S1. Clinicopathologic features (valid cases and percentages).

Variables		RCC	TCC	
			Proximal 2/3	Distal 1/3
		N. (%)	N. (%)	N. (%)
**Age**				
	Median (range)	70 (33–89)	68 (36–83)	68 (41–90)
	≤70 years	160 (52)	24 (60)	36 (63)
	> 70 years	154 (48)	16 (40)	21 (37)
**Sex**				
	Male	190 (41)	26 (65)	33 (58)
	Female	133 (59)	14 (35)	24 (42)
**Primary locations**				
	caecum	157 (49)	-	-
	ascending	166 (51)		
**ECOG PS**				
	0	209 (65)	31 (77)	35 (61)
	≥1	105 (32)	9 (23)	22 (39)
**Charlson Comorbidity Index**				
	≤8	204 (65)	17 (42)	31 (57)
	**>8**	111 (35)	23 (58)	23 (43)
**Tumor onset (*n* = 243)**				
	Anaemia	114 (47)	6 (22)	22 (49)
	Obstruction/Perforation	50 (21)	13 (46)	9 (20)
	Pain/fever/weight loss	79 (32)	9 (32)	14 (31)
**Surgery of primary tumor**				
	Yes	282 (87)	36 (90)	47 (83)
	Not	41 (13)	4 (10)	10 (17)
**AJCC TNM stage**				
	I	21 (7)	1 (3)	2 (3)
	II	82 (25)	13 (32)	11 (19)
	III	90 (28)	8 (20)	14 (25)
	IV	130 (40)	18 (45)	30 (53)
**Pathological Tumour size**				
	T1	7 (3)	0 (0)	3 (7)
	T2	24 (8)	1 (3)	0 (0)

T3	173 (64)	23 (77)	32 (71)
T4	67 (25)	6 (20)	10 (22)
**Pathological Node status**			
N0	119 (44)	14 (47)	14 (31)
N1	70 (26)	9 (30)	15 (33)
N2	80 (30)	7 (23)	16 (36)
**Mucinous Histology**	94 (29)	8 (22)	20 (36)
**Lymphovascular/Perineural invasion**			
Not	99 (45)	10 (42)	13 (34)
Yes	122 (55)	14 (58)	25 (66)
**Tumour differentiation**			
G1	15 (5)	2 (6)	1 (2)
G2	139 (49)	19 (54)	22 (45)
G3	129 (45)	14 (40)	26 (53)
G4	2 (1)	0 (0)	0 (0)
**Microsatellite Instability**			
MSS	78 (74)	6 (60)	17 (81)
MSI-H	28 (26)	4 (40)	4 (19)
**KRAS status**			
Wild-type	89 (44)	15 (48)	30 (73)
Mutant	113 (56)	16 (52)	11 (27)
**BRAF status**			
Wild-type	136 (79)	21 (88)	23 (68)
Mutant	36 (21)	3 (12)	11 (32)
**Adjuvant chemotherapy**			
**Yes**	111 (57)	11 (50)	19 (70)
**Not**	82 (43)	11 (50)	8 (30)
**Prevalent metastasis**			
Liver	115 (64)	15 (48)	24 (61)
Lung	19 (11)	6 (20)	3 (8)
Peritoneum	45 (25)	10 (32)	12 (31)

The total number could be different since some data are missed

© 2020 by the authors. Licensee MDPI, Basel, Switzerland. This article is an open access article distributed under the terms and conditions of the Creative Commons Attribution (CC BY) license (http://creativecommons.org/licenses/by/4.0/).
